# A Novel, Low-Cost Glaucoma Calculator to Identify Glaucoma Patients and Stratify Management

**DOI:** 10.1155/2022/5288726

**Published:** 2022-07-31

**Authors:** Daniel Laroche, Kara Rickford, Elise V. Mike, Liane Hunter, Ezekiel Ede, Chester Ng, John Douglas

**Affiliations:** ^1^New York Eye and Ear Infirmary, Icahn School of Medicine of Mount Sinai, New York, NY, USA; ^2^Advanced Eye Care of New York, New York, NY, USA; ^3^New York Medical College, Valhalla, NY, USA; ^4^Wilmer Eye Institute, Johns Hopkins Hospital, Baltimore, MD, USA; ^5^Montefiore Medical Center, Bronx, NY, USA; ^6^Mercer University School of Medicine, Savannah, GA, USA

## Abstract

Glaucoma is a leading cause of preventable blindness globally. Nearly, half of the patients who have glaucoma in the United States are unaware of their diagnosis, and this number is far greater in resource poor areas. The risk of progressive and irreversible loss of vision is decreased with an early diagnosis, and better access to treatment is vital to improve the visual outcome for patients. We therefore postulated that a minimally invasive, low-cost calculator used to predict the risk of glaucoma and inform the course of follow-up care will improve patient prognosis. We retrospectively examined data from 104 eyes of patients who underwent a complete ocular examination, visual field, and corneal pachymetry at Advanced Eye Care of New York (54 with glaucoma and 50 controls). Receiver operating curves (ROC) were utilized to determine the correct glaucoma classification rates of the Laroche glaucoma calculator (Range −3 to 18), a novel tool that combines age, intraocular pressure (IOP), and central corneal thickness (CCT) into a composite metric. Additionally, we compared the discriminatory power of this calculator to age, intraocular pressure (IOP), and central corneal thickness (CCT) separately. A score of greater than or equal to 6 on the Laroche glaucoma calculator (sensitivity 90.74%, specificity 64.00%, correct classification 77.88%) optimizes the accuracy of this tool. Compared to IOP (Area Under the Curve (AUC) = 0.72, chi^2^ = 4.21, *p*=0.04) and CCT (AUC = 0.53), chi^2^ 24.72 *p* < 0.001), the Laroche glaucoma calculator (AUC = 0.81) was significantly better at discriminating against glaucoma patients vs. controls. These results demonstrate that the Laroche calculator is a novel, effective tool for identifying glaucoma, and it may provide a low-cost risk stratification tool, particularly in areas with limited resources.

## 1. Introduction

Glaucoma is the second leading cause of blindness globally, with open-angle glaucoma affecting 74% of glaucoma patients in the US [1]. The prevalence of glaucoma in the U.S. increases with advancing age, affecting Black Americans aged 40 and older nearly three times more than White Americans [2]. Although common, glaucoma is difficult to detect and diagnose, and reported prevalence may underestimate the true number of affected individuals. Additionally, racial differences have been noted in the severity of glaucoma, and Black individuals are affected at an earlier age and have higher rates of blindness than their white counterparts [3]. Older age, particularly in non-Hispanic Blacks over the age of 40 and non-Hispanic whites over the age of 65, elevated intraocular pressure, myopia, and a family history of glaucoma are all well-established risk factors associated with glaucoma [1, 4]. As the population ages, the number of people with glaucoma globally is estimated to increase to 111.8 million by 2040 [5]. Glaucoma is especially prevalent among underserved populations with limited access to cataract surgery and is a leading cause of blindness in the African-American and Hispanic communities in the United States, Latin America, the Caribbean, and Africa [6]. Moreover, several other conditions are also thought to confer increased risk of glaucoma, including type and degree of refractive error, systemic hyper- and hypotension, vasospasm, migraine, pigmentary dispersion syndrome, pseudoexfoliation syndrome, obstructive sleep apnea syndrome, diabetes, medication interactions and side effects, the degree of exposure to intraocular and intracranial pressure elevations and fluctuations, genetics, and family history of the disease [7]. Early diagnosis and better access to treatment is vital to improve the visual outcome for patients with glaucoma and reduce the outcome of irreversible blindness.

The factors associated with the onset and progression of glaucoma have been extensively studied in large clinical trials, many of which have been sponsored by the National Institutes of Health (NIH) [8–11]. While other researchers have studied objective methods to assess the progression of glaucoma [12], there is an increasing need to assess the onset and presence of this disease [13]. This would allow patients to become more educated about the risks and receive earlier treatment to prevent the progression of glaucoma and blindness. Researchers in the Ocular Hypertension Treatment Study (OHTS) and the European Glaucoma Prevention Study (EGPS) addressed this challenge by determining the predictive factors of developing primary open angle glaucoma in patients with ocular hypertension [14]. These factors were narrowed down to older age, higher IOP, larger vertical cup to disc ratio, thinner central cornea thickness (CCT), and increased patterns of standard deviation. These five variables were then used to estimate the risk of ocular hypertension advancing to glaucoma within the next 5 years. This calculator is beneficial in alleviating the complexities of deciding to treat ocular hypertension by providing an individualized risk estimate for each patient versus standard observation [14–16].

Existing risk calculators are not without drawbacks. The calculators used by the OHTS and EGPS groups require a high level of expertise to visualize the optic nerve and interpret visual fields. This eliminates the potential use of a glaucoma calculator by nonphysicians in resource poor areas. Additionally, De Moraes et al. recognized the inability of this calculator to identify patients with glaucoma, as it primarily evaluates untreated ocular hypertension [12]. Other studies, including the Early Manifest Glaucoma Trial (EMGT) evaluated the statistical relationship between IOP and CCT in the progression of glaucoma, but revealed a less precise risk assessment and have not released an equation [9, 16]. The EPIC-Norfolk Eye Study examined the association between IOP and glaucoma in approximately 9000 patients over 7 years (2004–2011) [17]. In this study, 76% of patients with newly found glaucoma were found to have an IOP below 21 mmHg and would have been missed by their standard of screening. Furthermore, 10% of those without glaucoma had an IOP greater than 21 mmHg suggesting the potential for overdiagnosis and unnecessary treatment. Age and central corneal thickness were not factored in their screening methodology. Overall, these studies show the potential for under- or overtreatment due to challenges with risk assessment. The development of a risk calculator, as suggested by Mansberger et al., can greatly impact patient treatment and management of glaucoma [16].

## 2. Hypothesis

The goal of this study is to develop a minimally invasive, low-cost method to predict the risk of glaucoma and inform the course of follow-up care. Specifically, we hypothesized that 3 risk factors—age, intraocular pressure (IOP), and central corneal thickness (CCT)—can be used to create a composite score that would effectively stratify patients based on their risk of developing glaucoma. The score is verified based on OCT, cup-to-disc ratio, and/or visual field (VF) anomalies. Risk stratification with the use of this calculator would inform the future time course of follow-up.

### 2.1. Evaluation of the Hypothesis

To test the hypothesis, we developed a risk calculator to screen patients for glaucoma and those patients at higher risk for glaucoma without the need for a visual field or optic nerve examination as part of the initial screening process. Additionally, we tested the novel calculator using three powerful numerical risk factors to determine its performance and clinical applicability. We utilized a database of patients that had corneal pachymetry as part of a complete glaucoma evaluation at a private glaucoma practice. A total of 104 eyes from 52 patients were identified. The relevant data were analyzed.

## 3. Methods

### 3.1. Patients

We used retrospective data from established patients at Advanced Eye Care of New York. Informed consent was previously obtained for comprehensive eye examination. We followed the Declaration of Helsinki, and this study was approved by the Institutional Review Board of the New York Eye and Ear Infirmary of Mount Sinai.

All patients had previously undergone a complete ophthalmic examination, including slit-lamp biomicroscopy, gonioscopy, Goldmann applanation tonometry, ultrasound pachymetry, dilated stereoscopic examination, photography of the optic disc, optical coherence tomography, and standard automated visual field testing.

Established glaucoma was defined as the presence of glaucomatous optic neuropathy associated with glaucomatous visual field (VF) abnormalities. A glaucomatous VF was defined as the presence of a glaucoma hemifield test (GHT) outside normal limits and a pattern standard deviation (PSD) with *P* < 0.05 on at least two consecutive examinations. Inclusion criteria included having been completely evaluated for glaucoma in our office with tonometry, CCT, dilated fundus examination (DFE), OCT and visual field. Criteria for controls were not having a diagnosis of glaucoma with a normal DFE, OCT ONH and visual field.

### 3.2. Laroche Glaucoma Calculator Design

The Laroche Glaucoma Calculator was designed based on three known risk factors for glaucoma: age, intraocular pressure (IOP) and central corneal thickness (CCT). Each of these risk factors was designated as a numerical variable between −3 and +6 (Table 1). CCT was assigned in increasing numerical order as the risk of glaucoma increases with thinning of the cornea. All three have a linear relationship with the prevalence of glaucoma. The numerical point values corresponding to each of the three risk factors can then be added to determine low or high risk of glaucoma (Table 2).

### 3.3. Statistical Analysis

Demographic characteristics were compared between participants with a diagnosis of glaucoma and controls using the Kruskal–Wallis or Analysis of Variance (ANOVA) test for continuous variables and *χ*^2^ or Fisher exact test for categorical variables. Receiver operating curves (ROC) (Pepe, M. S. 2003. The Statistical Evaluation of Medical Tests for Classification and Prediction. New York: Oxford University Press.) were run to assess the discriminatory power, also known as area under the curve (AUC), and correct classification rates of the (1) Laroche glaucoma calculator, (2) IOP, (3) CCT, and (4) age. Additionally, the AUC of the Laroche glaucoma calculator vs. IOP, CCT and age were analyzed. A nonparametric approach was then used to compare the areas under two or more correlated receiver operating characteristic curves, as demonstrated by DeLong et al. [18]. A *p* value less than 0.05 was considered statistically significant. All analyses were performed using Stata, Version 15.0 (StataCorp, LP, Texas, USA).

## 4. Results

### 4.1. Baseline Characteristics

A diagnosis of glaucoma was present in 54/104 (52%) of participants' eyes, and the mean age (SD) of the sample was 65.4 (14.7). The glaucoma patients were older with a mean age (SD) of 71.4 (13.0) in glaucoma when compared with controls who had a mean age of 58.8 ((13.9); *p* < 0.0001). Glaucoma patients demonstrated a significantly higher IOP (mean (SD) 23.7 (7.4)) than controls (18.6 (4.1)); *p* < 0.0001 and greater median baseline cup to disc ratio (0.8 [0.68, 0.85] than controls (0.6 [0.5, 0.7]; *p* < 0.0001). Glaucoma patients also had a significantly lower OCT optic nerve head (mean (SD) 64.6 (15.0)) than controls (78.6 (15.8); *p* < 0.0001] and lower baseline VF [median (IQR) −8.74 [ −17.95, −4.18] than controls (−3.9 [−5.79, −1.2]; *p* < 0.0001), as shown in Table 3. The patients in this study were African American and Afro-Latino individuals from the population of the practice in Harlem, New York, and Southeast Queens, New York City.

### 4.2. ROC Analyses

**Figure 1:**
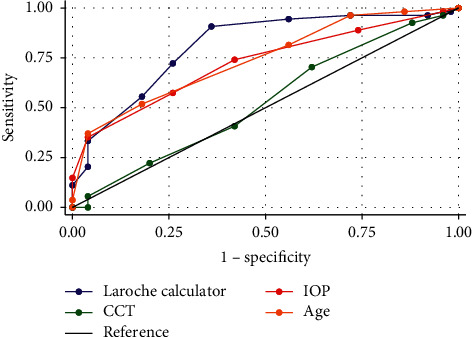
ROC curves from the laroche glaucoma calculator, IOP, CCT, and age. AUC for Laroche calculator = 0.82, AUC for IOP = 0.72, AUC for CCT = 0.53. AUC for age = 0.741. IOP = intraocular pressure, CCT = central corneal thickness.

The Laroche glaucoma calculator had a significantly greater diagnostic ability (AUC = 0.82) than IOP (AUC = 0.72, *p*=0.048), and CCT (AUC = 0.53, *p*=<0.0001), but not age (AUC = 0.741, *p*=0.174*p*=0.174) (Figure 1). Diagnostic properties and cut points of the Laroche glaucoma calculator, IOP, CCT, and age are demonstrated in Table 4. The average score in the control group was 3.96 (95% CI 3.29–4.62), while the average score in the patients with glaucoma was 6.72 (95% CI 6.08–7.36). A cut point of greater than or equal to 6 optimizes the accuracy of the Laroche glaucoma calculator (sensitivity (90.74%), specificity (64.00%), correct classification (77.88%)) IOP, CCT, and age demonstrated optimal cutpoints for accuracy of greater than or equal to 3 (Table 5), greater than or equal to 0 (Table 6), and greater than or equal to 4 (Table 7), respectively.

## 5. Discussion

We developed a low-cost glaucoma calculator to affordably screen patients for glaucoma. The use of this glaucoma calculator can allow nonmedical personnel to screen in underserved areas or remote areas and to refer the appropriate patients for a more intensive evaluation with medical personnel. This can provide earlier access to care for those with the greatest need. This can be particularly valuable in high person volume areas such as pharmacies, place of worship, and remote areas with limited resources by trained nonmedical personnel. Although IOP is a modifiable risk factor for glaucoma, IOP alone is not sufficient for screening for glaucoma. The addition of age and corneal thickness to create the novel Laroche calculator has created a more powerful low-cost method for screening for glaucoma. This also does not require dependency of family history information nor more expensive optic disc evaluation that has been shown to be more sensitive and specific. This is a vital addition in resource-poor areas and physician shortage areas for screening without the need of optic disc examination, OCT or VF that are more expensive aspects of the screening process. These resources can be reserved to further evaluate the higher risk patients determined from the calculator screening.

In addition to the risk calculators from the OHTS and EGPS groups, other risk assessments in ophthalmology include those developed by the Diagnostics Innovation Glaucoma Study (DIGS) [19] and a study conducted by Mansberger and Cioffi [15] to determine the probability of developing glaucoma following ocular hypertension. Clinicians have agreed that risk calculations have been both clinically useful and beneficial to the patients financially [16, 19–25].

Tele-glaucoma may close the gap between those who require screening and those who are able to get evaluated in-office, but the costs can be significant, and vans can cost over $200,000 in expense per year. A pachymeter and tonometer alone is much less expensive without the need for a physician, OCT, or visual field as part of the initial screening. High risk patients can be referred to a tertiary care center for further comprehensive evaluation and treatment. A patient who is at low risk for developing glaucoma with good vision can potentially be re-evaluated in 6 to 12 months without the need for a complete exam in an area with limited resources. This also provides an opportunity to educate persons at risk about the importance of cataract and glaucoma as they age. This is not meant to replace the experience and clinical judgment of an eye specialist who will need to confirm those high-risk patients who have been screened.

Similar to the Framingham, OHTS, and DIGS risk models, the results of our risk calculator should be interpreted with caution. The basic assumptions in our model were a linear pattern of glaucomatous presence with elevation of intraocular pressure and age. Patients with thinner corneas have a lifelong higher risk of glaucoma based on their corneal biomechanics [26]. The present model should be used for screening and patient education. We do not suggest that risk calculators be used as a substitute for clinical judgment, but rather as supplemental tool. This will be useful for low-cost population screening particularly in areas with limited resources. Additional studies need to be performed in a larger sample size and another data set. Further studies need to be done to further validate this with a larger and different populations. This calculator can also be studied and incorporated in artificial intelligence programs with newer technology that can perform both contact and noncontact tonometry and pachymetry for further validation with larger data.

## 6. Conclusions

To our knowledge, our report represents the first attempt to generate an affordable screening calculator to identify patients with glaucoma or those at high risk for glaucoma, based on three risk factors without the need for a comprehensive ophthalmic exam, fundus exam, and visual field testing. This calculator is very promising for screening particularly in resource-poor areas and to provide cost effective glaucoma screenings by trained nonphysician allied health professionals. Our prediction model demonstrated excellent discrimination in identifying patients with glaucoma with optic nerve damage and visual field loss, as well as those at high risk for glaucoma. Future studies need to be performed to further validate this new glaucoma calculator.

## Figures and Tables

**Table 1:** The Laroche glaucoma calculator: risk factors with corresponding numerical variables.

Points	−3	−2	−1	0	+1	+2	+3	+4	+5	+6
Age (years)	—	—	—	≤40	41–50	51–60	61–70	71–80	81–90	≥91
Mean IOP^*∗*^ (mmHg)	—	—	—	≤12	13–15	16–18	19–21	22–25	26–29	≥30
Mean CCT^*∗*^ (*μ*m)	>600	576–600	551–575	526–550	500–525	475–499	450–474	425–449	400–424	<400

^∗^mean IOP and mean CCTwere determined using three measurements per eye.

**Table 2:** The Laroche glaucoma calculator: determining glaucoma risk and management.

Glaucoma risk category	Total point (s)	Risk management
Low risk	0–5	Re-evaluation recommended in 1 year
High risk	6–18	Complete ophthalmic evaluation needed as soon as possible

**Table 3:** Demographics in glaucoma vs. control participants (*N* = 104).

	Total sample	Glaucoma (*N* = 54)	Controls (*N* = 50)	*p*-value
Age, mean (SD) (years)	65.4 (14.7)	71.4 (13.0)	58.9 (13.9)	<0.0001
CCT, mean (SD) (*μ*m)	531.9 (38.2)	530.5 (35.1)	533.4 (41.5)	0.70
IOP, mean (SD) (mmHg)	21.2 (6.6)	23.7 (7.4)	18.6 (4.1)	<0.0001
OCT ONH, mean (SD) 1	72 [59, 83]	64.6 (15.0)	78.6 (15.8)	<0.0001
Baseline VF, (MD) median [IQR] 2	−5.26 [−10, −2.8]	−8.74 [−17.95, −4.18]	−3.9 [−5.79, −1.2]	<0.0001
Baseline CD ratio, median [IQR] 3	0.7 [0.5, 0.8]	0.8 [0.68, 0.85]	0.6 [0.5, 0.7]	<0.0001

**Table 4:** Diagnostic accuracy and cut-off for the laroche glaucoma calculator.

Cut point	Sensitivity (%)	Specificity (%)	Correctly classified (%)
≥−1	100.00	0.00	51.92
≥0	98.15	2.00	51.92
≥2	98.15	4.00	52.88
≥3	96.30	8.00	53.85
≥4	96.30	28.00	63.46
≥5	94.44	44.00	70.19
≥6	90.74	64.00	77.88
≥7	72.22	74.00	73.08
≥8	55.56	82.00	68.27
≥9	33.33	96.00	63.46
≥10	20.37	96.00	56.73
≥11	11.11	100.00	53.85

**Table 5:** Diagnostic accuracy and cut-offs for intraocular pressure.

Cut point	Sensitivity (%)	Specificity (%)	Correctly classified (%)
≥−1	100.00	0.00	51.92
≥0	98.15	4.00	52.88
≥2	88.89	26.00	58.65
≥3	74.07	58.00	66.35
≥4	57.41	74.00	65.38
≥5	35.19	96.00	64.42
≥6	14.81	100.00	55.77

**Table 6:** Diagnostic accuracy and cut-offs for central corneal thickness.

Cut point	Sensitivity (%)	Specificity (%)	Correctly classified (%)
≥−3	100.00	0.00	51.92
≥−2	96.30	4.00	51.92
≥−1	92.59	12.00	53.85
≥0	70.37	38.00	54.81
≥1	40.74	58.00	49.04
≥2	22.22	80.00	50.00
≥3	5.56	96.00	49.04
≥5	0.00	96.00	46.15

**Table 7:** Diagnostic accuracy and cut-offs for age.

Cut point	Sensitivity (%)	Specificity (%)	Correctly classified (%)
≥0	100.00	0.00	51.92
≥1	98.15	14.00	57.69
≥2	96.30	28.00	63.46
≥3	81.48	44.00	63.46
≥4	51.85	82.00	66.35
≥5	37.04	96.00	65.38
≥6	3.70	100.00	50.00

## Data Availability

All data supporting the conclusions of this study have been included in this article.
